# The brain in motion–cognitive effects of simultaneous motor activity

**DOI:** 10.3389/fnint.2023.1127310

**Published:** 2023-05-25

**Authors:** Maren Schmidt-Kassow, Jochen Kaiser

**Affiliations:** ^1^Institute of Medical Psychology, Goethe University, Frankfurt, Germany; ^2^Department of Psychiatry, Psychosomatic Medicine and Psychotherapy, University Hospital, Goethe University, Frankfurt, Germany

**Keywords:** exercise, embodiment, physical activity, EEG, dual task

## Abstract

During the last 30 years, a large number of behavioral studies have investigated the effect of simultaneous exercise on cognitive functions. The heterogeneity of the results has been attributed to different parameters, such as intensity or modality of physical activity, and the investigated cognitive processes. More recent methodological improvements have enabled to record electroencephalography (EEG) during physical exercise. EEG studies combining cognitive tasks with exercise have described predominantly detrimental effects on cognitive processes and EEG parameters. However, differences in the underlying rationale and the design of EEG versus behavioral studies make direct comparisons between both types of studies difficult. In this narrative review of dual-task experiments we evaluated behavioral and EEG studies and discuss possible explanations for the heterogeneity of results and for the discrepancy between behavioral and EEG studies. Furthermore, we provide a proposal for future EEG studies on simultaneous motion to be a useful complement to behavioral studies. A crucial factor might be to find for each cognitive function the motor activity that matches this function in terms of attentional focus. This hypothesis should be investigated systematically in future studies.

## Introduction

For a long time, the effect of motor activity on cognitive processes has been studied asynchronously. Regular physical activity has been found to improve a variety of cognitive processes and to slow down cognitive aging (Stillman et al., 2020). However, in the last decade, increased research has addressed the effect of *simultaneous* motor activity on cognitive processes. This is considered “the natural form of thinking,” i.e., the functioning of the human brain is inherently linked to active exploration of the environment and thus motor activity (Clark, 1998; Wilson, 2002; Gramann et al., 2011). In this context, different methodologies have been used to assess cognitive processes during motor activity, i.e., behavioral measures as well as neuroimaging techniques. The former reveal outcomes such as reaction time, accuracy, or movement kinematics. Thus, the speed and accuracy of a response can provide information about the efficiency of cognitive processing, while movement kinematics can reveal the strategies used to perform a task. On the other hand, neuroimaging techniques [functional magnetic resonance imaging (fMRI), magnetoencephalography (MEG), and electroencephalography (EEG)] can be used to measure brain activity during motor tasks and to identify regions of the brain that are involved in specific cognitive processes. EEG and MEG can also reveal the temporal dynamics of cognitive processes during motor activity, such as the sequence of cognitive events that occur before and after a motor response.

However, all of these methods have limitations. Behavioral measures, while providing useful information about motor performance and cognitive processing, can be limited in their ability to capture the complexity and subtlety of cognitive processes. Neuroimaging techniques, while providing detailed information about brain activity, are limited in their application range. A meaningful analysis of MEG and fMRI data requires that the subjects sit physically still. This reduces the selection of movement tasks to very subtle motor activity, such as finger tapping. EEG on the other hand, allows more natural motor activity, however, one has to keep in mind potential challenges, including motion artifacts, sweat, and changes in scalp impedance. Motion artifacts can arise due to movement during exercise, which can introduce noise into the EEG signal. Sweat can also be a problem during exercise, as it can create electrical interference and alter the electrical properties of the scalp such as its impedance, which can affect the quality of the EEG signal. Despite these challenges, measuring EEG during exercise can provide valuable information about the neural processes underlying exercise performance and the effects of exercise on brain activity. For this reason, the current review aims to follow on a recent and comprehensive review on the behavioral outcomes of concurrent motor activity provided by Cantelon and Giles (2021), by comparing data from behavioral studies with data from EEG studies that have taken place under simultaneous movement.

In the course of this review it will become clear that most of the EEG and behavioral studies were carried out with different objectives, which makes a systematic comparison challenging. Behavioral studies were conducted with clear working hypotheses on how and when exercise affects cognitive processes. These working hypotheses differ markedly between studies. One group of hypotheses has focused primarily on the physiological relationship between movement and cognitive functions, whereas a second group of primarily psychological theories included assumptions about neuronal mechanisms. There are two influential physiological theories. (1) The arousal hypothesis, one of the earliest hypotheses on how exercise affects lower-level sensory and perceptual processes, is based on arousal theories (Yerkes and Dodson, 1908; Easterbrook, 1959). It predicts that increases in arousal and catecholamines alter performance on sensory and motor tasks in an inverted u-shaped manner (McMorris and Graydon, 1997): moderate, as opposed to both high- or light-intensity, exercise would lead to an improvement in cognitive functions [but see Vinck et al. (2015) for evidence that locomotion is not inherently linked to arousal]. (2) The hypofrontality theories, e.g., the “transient hypofrontality” (Dietrich, 2006), or the “reticular-activating hypofrontality” theory (Dietrich and Audiffren, 2011) were based on data from executive functions that are dependent on prefrontal cortex activity. They predict that these executive control processes benefit from simultaneous movement up to a certain intensity and/or duration, beyond which they collapse.

There are also two influential psychological models. The Attentional Resource Hypothesis (3) (Craik and Byrd, 1982) assumes that in dual task situations, one task is weighted more heavily than the other, and predicts that performance in a cognitive task will deteriorate with simultaneous movement [see Plummer et al. (2013) for an overview of different dual task scenarios]. According to the “posture first” strategy, humans should first ensure secure posture and then carry out the cognitive task with the remaining resources (Bloem et al., 2001). Thus, when the brain is too busy with the motor challenge [like an increase of neck muscle activity (Gramann et al., 2011), keeping posture, walking without stumbling, etc.], this should lead to reduced resources for the cognitive task. Finally, the entrainment theory (4) is based on evidence that motor activity leads to biological changes that are inherently rhythmical, such as brain activity, heart rate, or cardiorespiratory rate, and the human ability to synchronize body movements with external and internal rhythms (Thaut et al., 2005, 2014). For instance, external auditory rhythms lead to synchronization of body movements such as swaying, head nodding, clapping or dancing. In the framework of this theory, the rhythmic structure of movement should result in a template for information processing that facilitates encoding when information occurs in certain rhythmic phases (Jones and Boltz, 1989; Large and Jones, 1999).

For a long time, the scientific focus of the behavioral studies has been on the predictions of hypotheses (1) or (2). Hence, research on simultaneous exercise has focused on reaction times and accuracy data, or physiological data like hormonal changes or heart rate. However, recent methodological developments have enabled the measurement of neurophysiological processes during exercise (Debener et al., 2012; Jungnickel and Gramann, 2016; Kuziek et al., 2018; Radüntz and Meffert, 2019), which has led to a radical paradigm shift. Whereas about 15–20 years ago, EEG has been recorded almost exclusively in soundproof booths and with as little muscle activity as possible, an increasing number of recent studies have investigated how neurophysiological processes change with simultaneous movements.

Most behavioral studies have very systematically varied or controlled parameters such as duration, modality or cognitive process to examine one of the above mentioned hypotheses. In contrast, the main focus of the EEG studies has been on methodological developments including the reliable recording of event-related potentials, while reducing the above described artifacts. As a result, there are still important differences between behavioral and EEG studies, as the variability of the investigated cognitive processes as well as the duration and intensity of movement have been strongly limited in EEG research. Therefore, in this narrative review we first summarize findings from behavioral studies concerning the impact of simultaneous movement on cognition and then, in the second part, evaluate the additional contributions of EEG studies. The current work does not aim to evaluate the hypotheses described above, but rather to create an awareness of the problem of poor comparability between behavioral and EEG studies. Moreover, we will suggest some methodological aspects that should be taken into consideration in future studies so that EEG studies can make a valuable contribution to research on movement.

In summary, we aim to:

1) Give an overview on how simultaneous motor activity affects cognitive processes,
2) Discuss (contradictory) evidence from behavioral and EEG studies, and
3) Suggest recommendations and provide a proposal for future EEG studies on simultaneous motion to be a useful complement to behavioral studies, considering the issue of matching the type of motor activity to each cognitive process.

## Methods

Considerable research has examined the effect of simultaneous motor activity on a cognitive task. The focus of the present review is on experiments in which dual-task conditions included a cognitive task while healthy adults (18–50 years) were engaged in physical activity compared to a single-task condition, i.e., performing a cognitive task while being sedentary. The motion condition intervention required the continuous activation of large muscle groups. A total of 79 publications covering 88 studies were retrieved following topical key-term searches. Search terms included combinations of “acute,” “simultaneous,” “during,” “exercise,” “motor activity,” “physical activity” “movement,” “walking,” “running,” “cycling,” “pedaling,” “EEG,” “event-related potential,” “cognition,” “cognitive function,” “cognitive performance,” or cognitive tasks previously identified in reviews of this field [i.e., “oddball,” see Pontifex et al. (2019) for a list of cognitive tasks]. Sources of studies published through April 24, 2023 included Pubmed, Google Scholar, and reference lists from empirical reports and reviews. Experiments comparing different exercise intensities without a sedentary control condition were excluded. The purpose of the present selective review is to evaluate similarities and differences between behavioral and neurophysiological studies and to draw conclusions about the key open questions in motor-cognitive research. Our intent was not to conduct a systematic review or a meta-analysis but to provide a comprehensive state of the art of the field and make suggestions for future research. Throughout the review we use the following criteria from the American College of Sports Medicine guidelines for Exercise Testing and Prescription (Garber et al., 2011) to describe intensity categories of the reported experiments: very-light (i.e., <37% VO2max or <57% HRmax), light (37–45% VO2max or 57–63% HRmax), moderate (45–63% VO2max or 64–76% HRmax), vigorous (64–90% VO2max or 77–95% HRmax) and near-maximal to maximal (91% VO2max or 96% HRmax).

### Behavioral studies

Two comprehensive meta-analyses (Lambourne et al., 2010; McMorris and Hale, 2012) concluded that the effects of acute exercise on a cognitive task (measured either during or post-exercise) depend on the exact cognitive process, exercise intensity, exercise modality, and exercise duration. In sum, they found the most beneficial effects for speed of processing in executive functions at moderate exercise intensity (McMorris and Hale, 2012) and for cycling on a stationary bike (Lambourne et al., 2010), especially if exercise duration exceeded 20 min. Concerning exercise modality, they concluded that cycling outperforms treadmill running, since treadmill running requires complex motor and cognitive resources, i.e., posture, balance, and restricted movement execution, resulting in higher attentional demands than ergometer cycling. With these insights in mind, we have paid special attention to the modality and duration of the experimental paradigm and structured the review according to the cognitive processes investigated. In the current review, we included 60 studies that combined motor activity with cognitive tasks and compared a movement condition with a rest condition to evaluate the effect of simultaneous movement on a cognitive task. A total of 13 of them reported negative effects of simultaneous motor activity on cognitive functions, 34 reported positive effects on cognitive functions and 13 studies resulted in heterogeneous or null results.

In our review, we followed the approach of Cantelon and Giles (2021) and took a closer look at different subprocesses of executive function- (EF) and non-EF tasks. Here, EF included motor and cognitive inhibition, working memory and cognitive flexibility, and non-EF tasks included attention, information processing, motor speed, decision making, and long term memory [more comprehensive descriptions of all processes and associated paradigms can be found in Cantelon and Giles (2021), or Pontifex et al. (2019)]. In the current review we pooled studies on information processing and motor speed under the term “vigilance.” Furthermore, we focused on the different experimental durations used in the studies. For six studies, we were not able to determine the duration of the motor activity reliably. The remaining 54 studies were divided into four categories based on the experimental duration: (a) less than 15 min, (b) 15–30 min, (c) 31 to 45 min, and (d) more than 45 min. Almost half of the studies (*N* = 24) used paradigms that lasted 15–30 min, followed by short paradigms lasting less than 15 min (*N* = 17), while seven studies used paradigms with a duration from 31 to 45 min and six with more than 45 min (see Table 1).

**TABLE 1:** Summary of behavioral studies on simultaneous exercise during cognitive tasks.

Running number	References	Cognitive process	Exercise modality	Duration	Exercise intensity	Basic findings
1.	Bullock et al., 2015	Attention	Cycling	>45 min.	Light, moderate	Faster target detection during high-intensity cycling
2.	Giorno et al., 2010	Attention	Cycling	15–30 min.	Moderate, vigorous	Lower accuracy during exercise
3.	Huertas et al., 2011	Attention	Cycling	30–45 min.	Moderate, vigorous	No effects of moderate or vigorous cycling
4.	Pesce et al., 2003	Attention	Cycling	<15 min.	Moderate	Faster reaction times during moderate cycling
5.	Pesce et al., 2004	Attention	Cycling	<15 min.	Moderate	Faster reaction times during moderate cycling
6.	Radel et al., 2018	Attention	Cycling	>45 min.	Light, moderate	Faster reaction times during moderate intensity exercise
7.	Sanabria et al., 2011	Attention	Cycling	15–30 min.	Very light, moderate, vigorous	Faster reaction times during exercise
8.	Sanchis et al., 2020	Attention	Cycling	30–45 min.	Light, moderate	No effects of moderate or light low cycling
9.	Shields et al., 2011	Attention	Cycling	N.A.	Very light, light. Vigorous	Better performance for light exercise
10.	Travlos and Marisi, 1995	Attention	Cycling	N.A.	Light, moderate, vigorous	No effects of light, moderate or vigorous cycling
11.	Wohlwend et al., 2017	Attention	Running (treadmill)	N.A.	Light, moderate	Lower accuracy during exercise
12.	Yagi et al., 1999	Attention	Cycling	<15 min.	Moderate	Faster reaction times for moderate cycling
13.	Murali and Händel, 2022	Cognitive flexibility	Walking in a room	N.A.	N.A., self-paced	Better performance for unrestricted walking condition
14.	Oppezzo and Schwartz, 2014	Cognitive flexibility	Walking (treadmill)	<15 min.	Very light, light	Better performance for light exercise condition
15.	Tomporowski and Audiffren, 2014	Cognitive flexibility	Walking (treadmill)	<15 min.	N.A., walking at a preferred pace, walking at a faster pace	Faster response times and reduced local- and mixed-switch costs during treadmill walking at preferred pace
16.	Ando et al., 2011	Cognitive inhibition	Cycling	<15 min.	Light, moderate, vigorous	Faster reaction times during moderate exercise
17.	Davranche and McMorris, 2009	Cognitive inhibition	Cycling	15–30 min.	Moderate	Poorer response inhibition during exercise
18.	Davranche et al., 2009	Cognitive inhibition	Cycling	15–30 min.	Moderate	Faster reaction times during exercise
19.	Finkenzeller et al., 2019	Cognitive inhibition	Cycling	30–45 min.	Very light to moderate	Faster reaction times during exercise
20.	John et al., 2009	Cognitive inhibition	Walking (treadmill)/running	>45 min.	Very light	No difference between seated condition and treadmill walking
21.	McMorris et al., 2009	Cognitive inhibition	Cycling	15–30 min.	Moderate, vigorous	No difference between seated condition and moderate cycling
22.	Olson et al., 2016	Cognitive inhibition	Cycling	15–30 min.	Light, moderate	Faster reaction times during exercise
23.	Pontifex and Hillman, 2007	Cognitive inhibition	Cycling	<15 min.	Light	Poorer performance during light cycling
24.	Schmit et al., 2015	Cognitive inhibition	Cycling	15–30 min.	Vigorous	Lower accuracy during vigorous cycling
25.	Amico and Schaefer, 2020	Long-term memory	Running	N.A.	N.A.	No effect
26.	Frith et al., 2017	Long-term memory	Running (treadmill)	15–30 min.	Light to vigorous	No effect
27.	Miles and Hardman, 1998	Long-term memory	Cycling	<15 min.	Moderate	No effect
28.	Pyke et al., 2020	Long-term memory	Cycling	15–30 min.	Light, moderate, vigorous	Better performance during cycling at varying intensities
29.	Schmidt-Kassow et al., 2010	Long-term memory	Cycling	15–30 min.	Moderate	Better performance during moderate cycling
30.	Schmidt-Kassow et al., 2014	Long-term memory	Walking (treadmill)	15–30 min.	Light	Better performance during light treadmill walking
31.	Schmidt-Kassow et al., 2013a	Long-term memory	Cycling	15–30 min.	Moderate	Better performance during moderate cycling
32.	Silvers et al., 2018	Long-term memory	Cycling	15–30 min.	Very light, light, vigorous	No effect
33.	Ando et al., 2013	Motor inhibition	Cycling	<15 min.	Vigorous	Faster reaction times during vigorous cycling
34.	Joyce et al., 2009	Motor inhibition	Cycling	15–30 min.	Light	Faster reaction times during light cycling
35.	Komiyama et al., 2015	Motor inhibition	Cycling	15–30 min.	Moderate	Faster reaction times and higher accuracy during moderate cycling
36.	Komiyama et al., 2016	Motor inhibition	Cycling	15–30 min.	Moderate	Faster reaction times during moderate cycling
37.	Komiyama et al., 2017	Motor inhibition	Cycling	15–30 min.	Moderate	Faster reaction times during moderate cycling
38.	Smith et al., 2016	Motor inhibition	Running (treadmill)	<15 min.	Vigorous	Poorer performance during vigorous running
39.	Ando et al., 2008	Vigilance	Cycling	<15 min.	Moderate	Slower reaction times to peripheral visual stimuli during moderate exercise
40.	Arcelin and Brisswalter, 1999	Vigilance	Cycling	<15 min.	Moderate	Better performance during moderate cycling
41.	Arcelin et al., 1998	Vigilance	Cycling	<15 min.	Moderate	Better performance during moderate cycling
42.	Audiffren et al., 2008	Vigilance	Cycling	30–45 min.	Moderate	Faster reaction times for moderate exercise
43.	Brisswalter et al., 1997	Vigilance	Cycling	<15 min.	Very light, light, moderate, vigorous	Increased reaction times for exercise condition
44.	Chmura et al., 1998	Vigilance	Cycling	15–30 min. > 45 min.	Very light, moderate	Better performance during moderate cycling
45.	Collardeau et al., 2001	Vigilance	Treadmill and over-ground Running	>45 min.	Moderate	Better performance for vigorous exercise
46.	Davranche and Audiffren, 2004	Vigilance	Cycling	15–30 min.	Very light, moderate	Faster reaction times for moderate exercise
47.	Lambourne et al., 2010	Vigilance	Cycling	30–45 min.	Moderate	Better performance during moderate cycling
48.	McMorris and Graydon, 1997	Vigilance	Cycling	15–30 min.	Moderate, vigorous	Better performance during near-maximum cycling
49.	Paas and Adam, 1991	Vigilance	Cycling	<15 min.	Very light, moderate, vigorous	Worse perception during exercise
50.	Paas and Adam, 1991	Vigilance	Cycling	<15 min.	Very light, moderate, vigorous	Better decision performance during exercise
51.	Amico and Schaefer, 2021	Working memory	Walking	N.A.	Light	Poorer performance during walking
52.	Dietrich and Sparling, 2004	Working memory	Running (treadmill) and cycling	30–45 min.	Moderate	Poorer performance in exercise condition
53.	Komiyama et al., 2015	Working memory	Cycling	15–30 min.	Moderate	No significant effects
54.	Komiyama et al., 2016	Working memory	Cycling	15–30 min.	Moderate	No significant effects
55.	Komiyama et al., 2017	Working memory	Cycling	15–30 min.	Moderate	No significant effects
56.	Lambourne et al., 2010	Working memory	Cycling	30–45 min.	Moderate	No significant effects
57.	Lo Bue-Estes et al., 2008	Working memory	Running (treadmill)	15–30 min.	Vigorous	Poorer performance for vigorous running
58.	Quelhas Martins et al., 2013	Working memory	Cycling	<15 min.	Moderate	Better performance during moderate cycling
59.	Rattray and Smee, 2016	Working memory	Cycling	>45 min.	Light, moderate, vigorous	Better performance during moderate cycling
60.	Wang et al., 2013	Working memory	Cycling	15–30 min.	Light, moderate, vigorous	Lower accuracy during moderate compared to low intensity

### Executive functions

#### Cognitive inhibition

Cognitive inhibition refers to the ability to ignore irrelevant information or distraction and focus on relevant information in order to achieve a particular goal. Hence, it is the capacity to suppress or inhibit automatic or prepotent responses in order to respond appropriately to changing situational demands. Frequently used paradigms to study cognitive inhibition are the Stroop task (Stroop, 1935), the Simon task (Simon, 1969), the Flanker task (Eriksen and Eriksen, 1974) and the Go/No-Go task [motor inhibition (Logan et al., 1984)].

In the current review nine studies investigated the effect of motor activity on cognitive inhibition and six studies investigated motor inhibition with a Go/No-Go task. For cognitive inhibition, five studies showed null results or negative effects of exercise on flanker tasks (Pontifex and Hillman, 2007; McMorris et al., 2009; Schmit et al., 2015), a Simon (Davranche and McMorris, 2009) or a Stroop task (John et al., 2009). All experiments included visual paradigms and were primarily conducted on a stationary bike. While both Pontifex and Hillman (2007) and Davranche and McMorris (2009) compared moderate intensity with resting, Schmit et al. (2015) assessed vigorous exercise. The latter study compared the first three with the last 3 min of a 20-min vigorous exercise condition and both phases with a rest control condition. They found that performance was unaffected during the first 3 min, whereas there was an increased occurrence of fast impulsive errors during the final 3 min. Pontifex and Hillman (2007) provided evidence that approximately 12 min of moderate exercise resulted in reduced response accuracy for incongruent trials relative to rest. In Davranche and McMorris’s (2009) experiment, participants cycled for 21 min. Overall performance was better (faster reaction times with preserved accuracy) when the Simon task was performed simultaneously with exercise. However, response inhibition, measured by the Simon congruency effect, deteriorated during exercise. McMorris et al. (2009) compared rest with moderate and vigorous cycling while performing an Eriksen flanker task. Reaction times and number of errors were unaffected by the moderate condition, whereas performance declined during the vigorous condition. In contrast, very light-intensity treadmill exercise did not affect performance on a Stroop task compared to the seated condition (John et al., 2009).

Four studies found positive effects of exercise on visual Flanker paradigms (Davranche et al., 2009; Ando et al., 2011; Olson et al., 2016; Finkenzeller et al., 2019). They were all conducted on a stationary bike at various intensity levels. In all studies, moderate exercise intensity improved reaction times. Here, exercise duration varied between 2*15 min (Davranche et al., 2009), 25 min (Olson et al., 2016) and 45 min (Finkenzeller et al., 2019).

Interestingly, comparisons of different intensities reliably showed a superiority of moderate intensity over both vigorous or light intensity (McMorris et al., 2009; Olson et al., 2016; Finkenzeller et al., 2019). In agreement with the arousal hypothesis, moderate exercise thus seems to have a positive effect on cognitive inhibition. Furthermore, cognitive inhibition seems to benefit more from cycling compared to walking or running. Hence a combination of the parameters intensity and modality seems to be essential.

For the six studies that investigated the effect of exercise on motor inhibition all but one of the studies found positive effects of moderate intensity on either reaction times alone or on both reaction times and accuracy. Four studies used light to moderate cycling with a duration of 30 min (Joyce et al., 2009; Komiyama et al., 2015, 2016, 2017). One investigation assessed 10 min of vigorous cycling (Ando et al., 2013). The only study that reported a negative effect on reaction times and accuracy employed a 10-min bout of vigorous treadmill exercise (Smith et al., 2016). Hence, motor inhibition seems to benefit from simultaneous cycling at different intensity levels.

#### Cognitive flexibility

Cognitive flexibility refers to a person’s capacity to change their way of thinking and acting in response to varying circumstances, new information, or differing tasks. It involves the ability to switch between different tasks or mental states, shift attention, and adjust to new situations. Three studies investigated the effect of exercise on cognitive flexibility. They assessed walking of less than 15 min at light intensity either on a treadmill (Oppezzo and Schwartz, 2014; Tomporowski and Audiffren, 2014) or on the floor (Murali and Händel, 2022) and consistently found beneficial effects. In sum, short walking interventions increase cognitive flexibility.

#### Working memory

Working memory is a cognitive system relevant for temporarily holding and manipulating information. As such it is needed to carry out complex cognitive tasks, such as problem-solving, reasoning, decision-making, and learning. Paradigms used in the included studies were the Paced Auditory or Visual Serial Addition Task (Gronwall, 1977), Wisconsin Card Sorting Task (Berg, 1948), delayed response task (Malloy, 2011), Spatial Working Memory task (Della Sala et al., 1999), the Sternberg task, and the N-back task (Jaeggi et al., 2003).

In this review, ten studies investigated the effect of exercise on working memory. Here, most studies (*N* = 6) used 20- to 40-min exercise phases. While the majority of the cycling studies reported null results (Lambourne et al., 2010; Wang et al., 2013; Komiyama et al., 2015, 2016, 2017), Wang et al. (2013) as well as Dietrich and Sparling (2004) reported negative effects of moderate exercise on accuracy. Vigorous treadmill exercise (Lo Bue-Estes et al., 2008) and free but restricted walking at light intensity (Amico and Schaefer, 2021) resulted both in negative effects. All of the studies used visual paradigms except for Lambourne et al. (2010) who employed the paced auditory serial addition task (PASAT). Studies with positive effects on working memory were all conducted on a stationary bike. Quelhas Martins et al. (2013) applied the PASAT at moderate intensity, and the visual Sternberg paradigm at different intensity levels. For both experiments, the authors found beneficial effects on working memory performance at moderate intensity with an exercise duration of about 8 min. Rattray and Smee (2016) reported positive effects for reaction times in a speeded match task at moderate intensity with an exercise duration of 50–55 min.

In sum, results for working memory were heterogeneous but overall indicate no effect or an impairment of working memory by simultaneous exercise (80%).

For executive functions, we found a challenging pattern of results. There is a trend for a superiority of moderate intensity exercise and cycling in comparison to other intensities and walking/running, respectively. In contrast, cognitive flexibility seems to benefit from walking at light intensity levels.

### Non-executive functions

#### Attention

The term “attention” is used to describe the ability to selectively focus on relevant information while filtering out distractors. It is a fundamental process that underlies many aspects of perception, cognition, and behavior and is closely related to executive functions. There are different types of attention, including sustained attention, which means the ability to maintain focus over a prolonged period of time, and selective attention, which refers to the ability to focus on one stimulus or task while ignoring other stimuli.

Twelve studies investigated the effect of motor activity on attention. Here, two studies, which both applied a visual continuous performance task, reported negative effects on attentional processing, either on a stationary bike (Giorno et al., 2010) or on a treadmill (Wohlwend et al., 2017). However, Wohlwend et al. (2017) only compared performance between high-, moderate- and light-intensity running, but they did not include baseline levels in the statistics. Descriptive results, however, suggested that there was only a negative effect of moderate exercise on false alarm rates, whereas light- or high-intensity exercise did not affect performance compared to the sitting condition. Giorno et al. (2010) compared rest with 24 min of moderate and high-intensity cycling while performing the continuous performance test (CPT), which is a combination of a sustained attention and inhibition task. They found an increase of false alarm rates in both exercise conditions compared to resting. In contrast, three studies reported no effects of exercise on attention, specifically concentration (Travlos and Marisi, 1995), alertness, spatial orientating, and executive control (Huertas et al., 2011), or sustained attention (Sanchis et al., 2020). They all used cycling at various intensity levels and had experimental durations of around 35 min.

Seven studies reported positive effects of motor activity on attention. They all used cycling as exercise modality. Yagi et al. (1999) and Bullock et al. (2015) applied an oddball paradigm that addresses selective attention and have also measured EEG data, hence these studies will be discussed in the EEG studies section. The other five studies covered a wide range of paradigms, i.e., sustained attention (Radel et al., 2018), spatial attention (Sanabria et al., 2011), attentional focusing (Pesce et al., 2003, 2004) and attentional control (Shields et al., 2011). They also covered highly variable experimental durations from 5 min (Pesce et al., 2003, 2004) to 50 min (Radel et al., 2018). In sum, these studies found faster reaction times for moderate exercise intensity as compared to light intensity or rest.

Taken together, the majority of studies investigating attention applied cycling paradigms, which makes conclusions concerning exercise modality impossible. Moderate intensity levels seem to have an overall beneficial effect.

#### Vigilance

Vigilance refers to a state of being alert, watchful, and attentive. This requires cognitive resources to be allocated to the task of monitoring, and to be maintained over a prolonged period of time. Vigilance tasks involve the need to quickly identify and respond to specific stimuli.

Twelve of the behavioral studies investigated the effect of exercise on vigilance and motor speed. Most of the studies used cycling paradigms. Brisswalter et al. (1997) tested participants with a visual simple reaction time task and found detrimental effects of 14 min of cycling at different intensity levels (light to vigorous) on reaction times compared to rest. Ando et al. (2008) recorded reaction times to visual stimuli presented in the central portion and periphery of the visual field during 9 min of pedaling at moderate intensity versus sitting. They found that the ability to respond to visual stimuli presented in the periphery of the visual field was vulnerable to simultaneous exercise. Paas and Adam (1991) reported a negative effect on performance in a 7-min perception paradigm. The majority of studies (*N* = 9), however, found positive effects on reaction times at different intensity levels (light to vigorous). They all applied visual reaction time tasks. McMorris and Graydon (1997) and Lambourne et al. (2010) reported positive effects on performance during 40 min of moderate exercise and during a very short (<5 min) near-maximum condition, respectively. Arcelin et al. (1998) and Arcelin and Brisswalter (1999) showed that 10 min of moderate cycling led to reduced reaction times and reduced error rate. Davranche and Audiffren (2004) and Audiffren et al. (2008) also showed that both 20 and 35 min at moderate intensity reduced reaction times. Chmura et al. (1998) only included male subjects and obtained the same results for 20 and 60 min of simultaneous exercise. In a decision paradigm (Paas and Adam, 1991) also found beneficial effects for 7 min of light and vigorous exercise in their predominantly male sample. The study by Collardeau et al. (2001) was the only one using a running condition. Comparing rest with 90 min of vigorous exercise, they found a beneficial effect of exercise.

In sum, vigilance as operationalized by reaction times benefits from different intensity levels at different movement durations. Since walking/running studies were rare, a comparison between modalities is not possible.

#### Long term memory

Long Term Memory is the part of the memory system where information is maintained for a longer amount of time, ranging from a few minutes to several years. In this review, eight studies investigated the effects of simultaneous exercise on long term memory. The following four of them found no differences between active and resting conditions on memory performance. Two studies assessed cycling on a stationary bike at different intensity levels and durations (Miles and Hardman, 1998; Silvers et al., 2018), one used a treadmill running paradigm (Frith et al., 2017) and one used free running (Amico and Schaefer, 2020). One limitation might be that in two of these studies, encoding times were rather short, i.e., Miles and Hardman (1998) used a 3-min encoding time and Amico and Schaefer (2020) used a 9-min encoding time, whereas Frith et al. (2017), and Silvers et al. (2018) used encoding times of 15 and 20 min, respectively.

The remaining four studies used primarily cycling paradigms and found positive effects on long-term memory in the auditory (Schmidt-Kassow et al., 2010, 2013a) and visual domains (Pyke et al., 2020) across different intensity levels. One study used a treadmill paradigm with very light intensity levels (Schmidt-Kassow et al., 2014). All of these studies used exercise interventions of approximately 30 minutes’ duration. The direct comparison of different intensity levels suggested the strongest effects during exercise at a moderate intensity level (Pyke et al., 2020).

### Interim conclusion

When we compare the different activity durations that have been used in behavioral studies on simultaneous motor activity and cognition, we found that for the short paradigms (<15 min), two thirds of the studies reported beneficial effects across a variety of cognitive processes. Studies that assessed 15–30 min of physical activity showed a similar distribution, with 56% reporting beneficial effects versus 20% showing negative effects. Hence both short and longer paradigms resulted in a comparable percentage of positive effects. For studies with a duration of 30–45 min and longer, it was difficult to draw a conclusion given the low sample size.

Concerning modality, 61% of the 13 studies testing the effects of walking/running found negative effects or null results on cognitive performance. The remaining 48 studies tested the effect of cycling. Sixty percent of these investigations revealed positive results.

Overall, vigilance (75% positive effects), motor inhibition (83% positive effects) and cognitive flexibility (100%) seemed to benefit most from simultaneous activity. However, it has to be kept in mind that only a small number of studies investigated cognitive flexibility and motor inhibition. For attention, evidence is rather mixed (58% positive effects), while cognitive inhibition (44% positive effects) and especially working memory (20% positive effects) seemed to be attenuated by simultaneous motor activity. In summary, trends can be identified for more beneficial effects of shorter (≤30 min) than longer activity durations, for cycling on a stationary bike compared with walking or running, and for more basic information processing compared with functions including attention, cognitive inhibition or working memory.

### EEG studies

Over the course of the last 10 years, a variety of new EEG systems have been developed that allow to record brain activity during movement [see Radüntz and Meffert (2019) for a recent comparison]. As a result, an increasing number of studies have been published that have investigated cognitive processes in more naturalistic settings including simultaneous movement. So far, the results are heterogeneous. Here we review a total of 29 studies that combined motor activity with EEG acquisition (see Table 2).

**TABLE 2:** Summary of EEG studies on motor activity during cognitive tasks.

Running number	References	Cognitive process	Exercise modality	Duration	Exercise intensity	Basic findings
1.	Akaiwa et al., 2022	Attention	Cycling	<15 min.	N.A.: optimal cadence, 30% faster than optimal cadence, and 30% slower than optimal cadence	P3 amplitude decreased under motorically active conditions.
2.	Bullock et al., 2015	Attention	Cycling	>45 min., divided into segments of 20 and 10 min.	Light, high	No effect for P3 amplitude but reduced P3 latency for exercise conditions. Faster reaction times for high intensity cycling.
3.	Chen et al., 2022a	Attention	Walking (corridor)	15–30 min., segmented into blocks of 4.5 min.	Very light	Increased N1, no effect for N2pc or alpha lateralization.
4.	Chen et al., 2022b	Attention	Walking (corridor)	15–30 min., segmented into blocks of 4.5 min.	Very light	Decreased pre-stimulus alpha and increased N1, increased post-stimulus alpha in the foveal area.
5.	Conradi et al., 2016	Attention	Cycling	<15 min.	Very light to light	Increased P3 during synchronized cycling, but not for random cycling.
6.	Cortney Bradford et al., 2019	Attention	Walking (treadmill)	15–30 min.	N.A.: unloaded vs. loaded rucksack with a load of up to ∼40% body weight	P3 amplitudes were decreased during walking compared to rest.
7.	De Vos et al., 2014	Attention	Walking outdoor	<15 min.	Very light to light	P3 amplitude decreased during walking compared to rest.
8.	Debener et al., 2012	Attention	Walking outdoor	<15 min.	Very light to light	P3 amplitude decreased during walking compared to rest.
9.	Gramann et al., 2010	Attention	Walking/running (treadmill)	15–30 min.	N.A.: slow, medium, fast	No effect for N1 nor P3 amplitude.
10.	Kuziek et al., 2018	Attention	Cycling	<15 min.	Very light	No significant effect for in MMN or P300
11.	Ladouce et al., 2019	Attention	Walking (treadmill and corridor)	<15 min.	Very light to light	P3 amplitude is reduced for treadmill walking compared to standing. P3 amplitude is reduced for hallway walking and being moved in a wheelchair compared to treadmill walking
12.	Reiser et al., 2019	Attention	Walking outdoor	15–30 min.	Very light to light	P3 amplitudes were decreased for during walking compared to rest.
13.	Scanlon et al., 2017	Attention	Cycling	<15 min.	Light	No significant effects.
14.	Scanlon et al., 2020	Attention	Walking outdoor	<15 min.	Very light to light	P3 amplitudes were decreased during walking compared to rest.
15.	Schmidt-Kassow et al., 2019	Attention	Cycling	<15 min.	Very light to light	Increased P3 during synchronized cycling, but not for random cycling.
16.	Schmidt-Kassow et al., 2013b	Attention	Cycling	<15 min.	Very light to light	Increased P3 during synchronized cycling, but not for random cycling.
17.	Shaw et al., 2018	Attention	Walking (treadmill)	<15 min.	Very light to light	N1 and P3 amplitudes decreased during walking.
18.	Yagi et al., 1999	Attention	Cycling	<15 min.	Moderate	Faster reaction times and decreased P3 latencies for exercise condition but decrease in P3 amplitude and increase in error rates.
19.	Zink et al., 2016	Attention	Cycling free and fixed	<15 min.	N.A.	Decreased P3b amplitude in free biking compared to rest, but no effect between stationary cycling and rest.
20.	Reiser et al., 2021	Cognitive flexibility	Walking Outdoor	15–30 min.	Very light to light	Pre-target CNV, P2, N2, and P3 decreased in walking.
21.	De Sanctis et al., 2014	Cognitive inhibition	Walking (treadmill)	15–30 min.	N.A.: self-paced and briskly	P3 and N2 amplitude decreased during both walking conditions compared to rest. No significant behavioral effects.
22.	Olson et al., 2016	Cognitive inhibition	Cycling	15–30 min.	Light, moderate	Increased P3b amplitudes and decreased reaction times for both exercise conditions relative to rest.
23.	Pontifex and Hillman, 2007	Cognitive inhibition	Cycling	<15 min.	Moderate	Better performance during moderate cycling but larger N1/N2/P3 amplitudes as well as increased latencies.
24.	Straetmans et al., 2022	Cognitive inhibition	Walking (floor)	15–30 min.	N.A.: preferred speed	Poorer Performance during walking. No significant effects for P3.
25.	Torbeyns et al., 2016	Cognitive inhibition	Cycling	15–30 min.	Light	Faster reaction times for exercise condition but no effect on P3 amplitude or latency.
26.	Benjamin et al., 2018	Vigilance	Walking (treadmill)	<15 min.	N.A.: briskly	No effects on behavior and SSVEP
27.	Cao and Händel, 2019	Vigilance	Walking (floor)	15–30 min., divided into blocks of 2 min. each	N.A.: slowly, or normal speed	Alpha power decreased during walking compared to rest.
28.	Protzak et al., 2021	Vigilance	Walking (corridor)	<15 min.	Very light to light	Faster reaction times for rest condition. P1 and P3 amplitude decreased and latencies increased during walking compared to rest.
29.	Vogt et al., 2015	Vigilance	Cycling	<15 min.	Light	No significant effects.

There are striking methodological differences between the behavioral studies reported above and the EEG work. First, EEG paradigms were usually shorter than activity phases in behavioral studies, i.e., in more than half of the paradigms used, participants were physically active for less than 15 min. These investigations reported improved event-related potential (ERP) parameters during movement. Second, more than half of the studies (16) used walking paradigms, whereas only 20% of the behavioral studies investigated walking. Here, only one study found a positive effect of walking (Cao and Händel, 2019) and two studies found mixed effects (Chen et al., 2022a,b). On the other hand, out of the 13 studies that tested the effect of cycling, only two studies found detrimental effects of exercise, while the majority revealed mixed or positive results.

Hence we argue that the higher percentage of negative effects on cognition in EEG studies might be mainly driven by the motor modality (i.e., walking) and maybe partly by differences in the duration of activity. In the following we want to look more closely at the different cognitive processes.

### Executive functions

#### Cognitive inhibition

Three out of the five studies on inhibition found mixed results (Pontifex and Hillman, 2007; Torbeyns et al., 2016; Straetmans et al., 2022), one found a negative effect (De Sanctis et al., 2014), and one found a positive effect (Olson et al., 2016). Studying a visual stroop task on a stationary bike, Torbeyns et al. (2016) found faster RT during the 4-min cycling condition (total cycling duration was 30 min), but no effects for accuracy or any of the measured ERPs, i.e., N200, P300 or N450. Pontifex and Hillman (2007) used a visual flanker task on a stationary bike (6 min) at moderate intensity. The results were rather mixed with reduced accuracy (as mentioned above) but enhanced P300 and N200 amplitudes. On the other hand, latencies of both components were prolonged for cycling compared to sitting. Straetmans et al. (2022) conducted an auditory competitive speaker paradigm while subjects were either walking in a public cafeteria (15 min) or sitting. They found higher accuracy levels for the sitting condition but no differences in the P300 component.

De Sanctis et al. (2014) studied a Go/Nogo task (i.e., motor inhibition) on a treadmill and found reduced N2 and P3 amplitudes but no behavioral effects when comparing about 16 min of self-paced walking or “walking briskly” with a rest condition. In contrast, Olson et al. (2016) found that 30 min of moderate and light cycling resulted in larger P300 amplitudes in a visual Flanker task, which was in line with the reported decrease in reaction times. In summary, the heterogeneous pattern of effects of simultaneous movement on inhibition processes found in EEG studies to some extent mirrors the findings from behavioral investigations.

#### Cognitive flexibility

Reiser et al. (2021) studied cognitive flexibility by applying an auditory task switching paradigm during 15 min of outdoor walking. They reported no impact on behavioral performance but found convergent negative effects on electrophysiological parameters, i.e., pre-target CNV as well as P2, N2, and P3 amplitudes decreased for the walking condition.

### Non-executive functions

#### Vigilance

Four EEG studies (Vogt et al., 2015; Benjamin et al., 2018; Cao and Händel, 2019; Protzak et al., 2021) investigated the influence of motor activity on vigilance. Three of the studies used walking paradigms and found mixed results. While Protzak et al. (2021) reported negative effects of 12 min of walking on reaction times as well as P300 amplitude and latency. Cao and Händel (2019) found that 16 min (separated in blocks of about 2 min each) of free walking improved behavioral measures of peripheral compared to central visual processing. This was accompanied by a decrease in alpha power. They argued that walking triggers an attentional shift toward peripheral input. Benjamin et al. (2018), however, provided evidence that 9 min of brisk treadmill walking had no effect on contrast sensitivity or surround suppression. The only cycling study compared sitting with light-intensity cycling (5 min) and found no significant differences between conditions neither for behavioral outcomes nor for the N200/P300.

#### Attention

The majority of EEG studies (*N* = 19) investigated the effect of simultaneous motor activity on attention processes and analyzed primarily the P300 followed by the N1/P1 and N2/P2 components as well as mismatch negativity (MMN). 8 studies found negative effects, 3 found positive effects, 8 found mixed effects. Except for four studies (Shaw et al., 2018; Reiser et al., 2019; Chen et al., 2022a,b), all studies used an oddball paradigm.

The three studies reporting positive effects of activity were all conducted in our laboratory with participants cycling on a stationary bike at very light intensity, during which they listened to auditory stimuli (Schmidt-Kassow et al., 2013b,^2019^; Conradi et al., 2016). Participants pedaled for 10 min in each condition. We found higher P300 amplitudes in response to rhythmically presented tones or syllables when subjects synchronized with stimulus presentation compared to sitting still.

Half of the studies with mixed results also used pedaling [3 auditory and 1 visual (Bullock et al., 2015; Zink et al., 2016; Scanlon et al., 2017; Kuziek et al., 2018)], while two assessed walking on a treadmill [1 auditory and 1 visual (Gramann et al., 2010; Ladouce et al., 2019)] and the other two applied a visual, free walking paradigm (Chen et al., 2022a,b). Comparing 20 min of light or high-intensity exercise to rest, Bullock et al. (2015) found no modulation of the P300 amplitude, but earlier P300 peak latencies. Furthermore, they found faster reaction times for high-intensity cycling. Zink et al. (2016) reported no behavioral data and found no differences in P300 amplitude or latency between 12 min of cycling at light intensity and sitting. Scanlon et al. (2017) investigated the effect of approximately 4 min of light-intensity cycling compared to sitting. They found no significant differences between the conditions for the P300. Similarly, Kuziek et al. (2018) found no differences in MMN or P300 after six minutes of very light-intensity cycling in comparison to a sitting condition.

For the walking studies, Gramann et al. (2010) asked participants to walk or run for twenty minutes at different speeds while performing a visual oddball task. They found no influence of walking speed on P300 or N100 amplitude. No behavioral performance has been reported. Ladouce et al. (2019) found lower accuracy rates during walking in a hallway, which was accompanied by a reduced P300 amplitude. In a very elegant second experiment, they systematically investigated how locomotion and varying visual inputs contributed to this effect. They compared 5 min of walking in a hallway, walking on a treadmill, being moved in a wheelchair and standing still. Interestingly, there were no differences between walking in a hallway and being moved in a wheelchair. Furthermore, they reported a larger P300 during walking on a treadmill compared to the hallway condition. However, the P300 amplitude in the standing still condition was still higher than during treadmill exercise.

The visual distraction paradigms conducted by Chen and colleagues (Chen et al., 2022a,b) reported mixed effects on visual attention. They compared 20 min of free walking with 20 min of standing (both divided into shorter experimental blocks). Chen et al. (2022a) found that early sensory processing (N1) was increased during movement, whereas subsequent processing steps in later time windows were unaffected by simultaneous motor activity (no effect for post-stimulus alpha power or P300). Chen et al. (2022b) suggested that walking results in a general state change that is indicated by reduced pre-stimulus alpha power and enhanced N1 amplitude. Furthermore, post-stimulus alpha power showed that the visual input in the foveal area was less processed than in peripheral areas while walking.

In sum, only 15% of the EEG studies on attention reported positive effects of simultaneous motor activity, while in the behavioral studies, the proportion was 58%. As the most commonly used paradigms included free walking, this may be a relevant factor that modulates the effect of simultaneous motor activity on the P300 in an oddball paradigm. In fact, the majority of studies with negative effects were free (outdoor) walking studies [*N* = 4 (Debener et al., 2012; De Vos et al., 2014; Reiser et al., 2019; Scanlon et al., 2020)], two other walking studies used a treadmill (Shaw et al., 2018; Cortney Bradford et al., 2019) and the remaining two were pedaling studies [visual: Yagi et al. (1999), tactile: Akaiwa et al. (2022)]. Most active conditions lasted for 10 min at maximum, except for Akaiwa et al. (2022), where one condition lasted about 15 min, and Cortney Bradford et al. (2019) and Reiser et al. (2019) where subjects walked on a treadmill for 1 h. All of the studies with negative effects on attention reported a decreased P300 amplitude in response to the locomotion condition as compared to the sitting or standing conditions.

## Discussion

**FIGURE 1:**
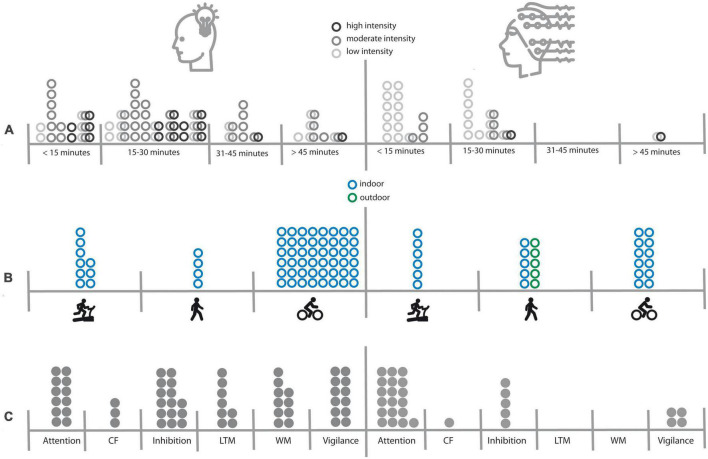
Overview of experimental factors that have been investigated in behavioral (left side) and EEG (right side) studies. Panel **(A)** illustrates the distribution of experimental durations and intensity levels. Interconnected rings symbolize that different intensities were compared in a study. Panel **(B)** illustrates the distribution of types of motor activity (walking/running on a treadmill, free walking/running, cycling) and indoor versus outdoor settings. Panel **(C)** illustrates the distribution of cognitive processes under investigation. CF, cognitive flexibility; LTM, long-term-memory; WM, working memory.

In sum, the present review revealed that EEG studies on simultaneous motor activity during cognitive tasks were conducted with a different focus than behavioral studies, making it difficult to compare both types of investigations. While behavioral studies were mainly theory-driven and hence have carefully controlled or justified the experimental durations, the cognitive processes, and the types of activity (please see Figure 1 for an illustration), most of the EEG studies focused more on methodological aspects, such as the reliability of ERPs in an indoor or outdoor setting, handling of noise and artifacts, or testing of mobile EEG systems. Hence, the most frequently used paradigm was the oddball paradigm to investigate the effect of motor activity on the reliably elicited and robust P300 component. However, even for the most intensively studied cognitive process, i.e., attention, there was only little variation in the duration, modality or intensity of motor activity (see Figure 1), making it difficult to draw meaningful conclusions from the EEG studies in comparison to the behavioral studies. This is regrettable insofar as behavioral studies have shown very mixed results with respect to attention processes. Here simultaneous EEG measurements could have helped to understand why different paradigms lead to different results. Concerning the parameter duration, behavioral studies on attention have covered a wide range of experimental durations from less than 15 min to more than 45 min. In the EEG studies, all but one (Bullock et al., 2015) of the experiments used block durations of less than 15 min, making it impossible to draw conclusions on how duration affects EEG results. Concerning the parameter modality, only one behavioral study that investigated attention used a running paradigm [on a treadmill (Wohlwend et al., 2017)], while 50% of the EEG experiments were walking studies, half of which used a free walking paradigm (in a hallway or outdoors). Especially free walking may result in additional cognitive load, since subjects have changing visual inputs and have to avoid obstacles, or may feel observed by people passing by. All these factors, which increase the cognitive load of the active condition, were not present in a sitting condition, making it impossible to conclude that motor activity *per se* reduces resources for a cognitive task. Lastly the factor intensity was also very heterogeneous across studies. While behavioral studies on attention covered all intensity levels from light to high intensity with a focus on moderate intensity, the prevailing majority of EEG study used light intensity levels only.

Hence, we encourage future EEG studies to restrict data collection to an indoor environment and compare different motor modalities under comparable conditions, i.e., cycling on a stationary bike versus walking on a treadmill versus sitting in the same room. This would ensure that external variables such as room temperature, traffic, visual input and environmental noise can be kept constant across experimental conditions. Furthermore, the comparison of different experimental durations and exercise intensities may provide meaningful insights into when and how motor activity supports or impairs a cognitive process. Admittedly, these comparisons are much more difficult in EEG than in behavioral studies, as the participants may sweat with increasing duration of the experiment or at higher intensities, which prevents a useful analysis of the EEG data. Hence, increases in experimental durations and higher intensity levels have to be carried out after careful consideration. Taking these challenges into account, EEG data nicely complement behavioral data. One of the advantages of EEG is its high temporal resolution. Thus, ERPs provide a continuous measure of processing between a stimulus and a response, making it possible to determine which stages are affected by a specific experimental manipulation. Another advantage over behavioral measures is that they can provide a measure of stimulus processing even when there is no difference in behavioral measures. Thus, EEG/ERP measurements combined with behavioral parameters may provide a comprehensive picture on when and how motor activity affects cognition. This also includes an aspect that has not been addressed in the current review, i.e., the electrode setup. Usually EEG studies use caps with 32–128 implemented electrodes. Most studies, however, select electrodes for analysis based on previous studies. This is hypothesis-driven and good scientific practice. However, these previous studies were typically conducted in restrictive sitting conditions where participants have been instructed to move and blink as little as possible. Hence it is conceivable to that simultaneous motor activity results in a different ERP topography. Therefore, we would suggest to use cluster-based permutation approaches instead of pre-defined region-of-interest contrasts to analyze ERPs under motion. One process that has not yet been studied with simultaneous EEG is long-term memory. This is regrettable, as behavioral studies have provided a very heterogeneous picture, i.e., half of the studies each have reported beneficial or negative effects, respectively. Hence, simultaneous EEG might shed more light on this process and how it unfolds across time, both on the millisecond- and on a minute-level. Thus applying EEG could enable us to disentangle whether performance differences are driven by differences at the encoding or retrieval level and how this is affected by experimental duration. As such, additional EEG data might be particularly useful for those processes that have previously provided a heterogeneous picture.

In summary, an impressive amount of work has been performed to make EEG measurements during both indoor and outdoor motion possible (Kranczioch et al., 2014) and to remove motion artifacts with elaborate algorithms to obtain reliable ERPs (Debener et al., 2012; De Vos et al., 2014; Kuziek et al., 2017, 2018; Radüntz and Meffert, 2019; Blum et al., 2020; Jacobsen et al., 2021). We are convinced that it is now time to move on to the next level, i.e., to address new, theory-driven questions in order to explain the heterogeneous picture that emerges from the behavioral studies on motion and cognition through the temporally high resolution of the EEG.

In the final part of our review we would like to suggest how simultaneous movement can be investigated in future studies and which factors lead to different results and thus to different conclusions. As already mentioned in the introduction, we assume that humans are *per se* moving creatures, i.e., phylogenetically, cognitive processes have always been accompanied by movement or served to plan movements. We therefore interpret movement as a natural state and accordingly would not classify it as an additional task in the same way as we would not classify seeing or hearing as additional tasks. In our view, movement only requires additional cognitive resources when it does not match the cognitive process under investigation.

The majority of EEG studies has shown that simultaneous movement interferes with cognitive processes, i.e., it resulted in decreased ERP components compared to sitting. Based on these results, it is reasonable to conclude that simultaneous movement leads to overload, as formulated in the Attentional Resources Hypothesis. According to the “posture first” strategy, humans should first ensure secure posture and then to carry out the cognitive task with the remaining resources (Bloem et al., 2001). However, the way we study simultaneous movement in the laboratory is very restrictive. In the case of ergometers, the movement execution is dictated by limitations imposed by the device. The advantage, on the other hand, is that visual and auditory inputs are controlled. When paradigms use free movement, it is not restricted in execution, but subjects have to stick to a certain route and pace. Both of these aspects are not found in a natural setting, where we automatically adapt our motor output to the current cognitive process. For example, if we want to solve a problem, most people would agree that walking or jogging in the forest will help them. However, when it comes to memorizing vocabulary, many prefer to walk up and down in a familiar room, facing the floor rather than looking around. This means that, depending on the form of movement, our attention is directed more toward the surroundings or toward a point, which can be helpful in different ways depending on the cognitive process. Hence, if we combine a restrictive movement with a cognitive process in our experimental design, we may wrongly conclude that simultaneous motor activity has detrimental effects on the cognitive process. This line of argumentation has been supported by recent evidence on the effects of motor restrictions on divergent thinking for different movement states (Murali and Händel, 2022). They reported a movement state-independent effect of restriction on creative thinking, i.e., both unrestrained walking (free walking instead of walking a pre-defined path) and unrestrained sitting (participants sat comfortably and faced the room while in the restricted sitting condition, they sat at a fixed distance from a computer screen with a fixation cross at the center) improved cognitive processing. The authors attributed the difference between restricted and unrestricted movement to a wider focus of attention for the latter. This interpretation is compatible with the observed stronger processing of peripheral stimuli during free walking (Cao and Händel, 2019; Chen et al., 2022b). It remains an open question whether this concept is applicable to other cognitive processes, too. For instance, in a selective attention paradigm, such as the frequently used oddball paradigm, a small attentional focus is useful to solve the task. As such, free walking (the most frequently used paradigm) may be a non-matching movement condition since it is associated with a broader attentional focus which in turn leads to poorer selective attention performance. This idea was supported by Chen et al. (2022b) who showed that free walking led to a more vigilant state (as indicated by decreased pre-stimulus alpha power and increased N1 component), but to reductions of selective attention (decreased post-stimulus P300).

On the other hand, in line with the entrainment hypothesis (4) described in the introduction [please see Tomporowski and Qazi (2020) for further discussion], motor synchronization should result in a narrower attentional focus. Thus, exercising on an ergometer may help to narrow attention (a) because of a stationary movement condition, and particularly if (b) motor activity is executed in synchrony with the incoming stimuli. This was the case in the oddball paradigms applied in our experimental setup (Schmidt-Kassow et al., 2013b,^2019^; Conradi et al., 2016). The same turned out to be true for behavioral long-term memory paradigms. Here, in the majority of the studies with positive effects, motor synchronization with the incoming stimuli was possible (Schmidt-Kassow et al., 2010, 2014, Komiyama et al., 2017). Future studies should try to clarify whether synchronization was actually the mechanism that led to a narrowed attentional focus, or which other parameters (restricted vs. free motor activity, exercise intensity, motor modality, cognitive process under investigation) may have contributed to the effect.

Let us sketch a few examples: For inhibition paradigms such as the Flanker paradigm, we would predict a negative effect of walking, since walking should result in a broader attentional focus, while solving the Flanker task requires a narrow visual attentional focus. Therefore, we would expect inhibition processes to benefit more from simultaneous cycling on a stationary bike, which is supported by the behavioral findings on cognitive inhibition reported above. On the other hand, cognitive flexibility benefited more from walking, which goes along with a broader attentional focus.

Studies included in our review have shown that working memory is impaired by walking or running, but unaffected or improved by stationary cycling. This is in contrast to transient hypofrontality models which propose that exercise shifts resources to areas required to monitor and control movements, resulting in working memory impairments during simultaneous exercise. However, the results are in line with the prediction that walking/running broadens the attentional focus while cycling on a stationary bike narrows the attentional focus.

Next to the effect of motor modality on the attentional focus, motor synchronization might be a key parameter to narrow the focus. This argumentation is supported by non-motor data from Plancher et al. (2018) who found that working memory performance was boosted by temporal regularities. In line with the entrainment hypothesis, they argued that participants synchronize their attention to the externally given beats, which leads to more attentional resources at certain time points and hence a better working memory performance. Future research should investigate whether this reported narrowing of the attentional focus by stimulus regularity can be further enhanced by synchronized motor activity on a stationary bike, resulting in increased working memory performance.

In sum, we propose to consider movement as a cognitive state, which in principle supports brain processes, as long as it is appropriate for the specific task. Therefore, future studies should take the into account whether the motor activity under investigation is ecologically relevant or not and this ecological relevance should be considered (next to intensity, duration, modality and task) for the interplay of motor activity and cognition.

## Author contributions

MS-K performed the literature search. MS-K and JK drafted the manuscript. Both authors contributed to manuscript revision, read, and approved the final manuscript.
